# Mining statistically-solid *k*-mers for accurate NGS error correction

**DOI:** 10.1186/s12864-018-5272-y

**Published:** 2018-12-31

**Authors:** Liang Zhao, Jin Xie, Lin Bai, Wen Chen, Mingju Wang, Zhonglei Zhang, Yiqi Wang, Zhe Zhao, Jinyan Li

**Affiliations:** 1 Precision Medicine Research Center, Taihe Hospital, Hubei University of Medicine, Shiyan, China; 2 School of Computing and Electronic Information, Guangxi University, Nanning, China; 3 Advanced Analytics Institute, Faculty of Engineering & IT, University of Technology Sydney, NSW 2007, Australia

**Keywords:** Error correction, Next-generation sequencing, *z*-score

## Abstract

**Background:**

NGS data contains many machine-induced errors. The most advanced methods for the error correction heavily depend on the selection of solid *k*-mers. A solid *k*-mer is a *k*-mer frequently occurring in NGS reads. The other *k*-mers are called weak *k*-mers. A solid *k*-mer does not likely contain errors, while a weak *k*-mer most likely contains errors. An intensively investigated problem is to find a good frequency cutoff *f*_0_ to balance the numbers of solid and weak *k*-mers. Once the cutoff is determined, a more challenging but less-studied problem is to: (i) remove a small subset of solid *k*-mers that are likely to contain errors, and (ii) add a small subset of weak *k*-mers, that are likely to contain no errors, into the remaining set of solid k-mers. Identification of these two subsets of *k*-mers can improve the correction performance.

**Results:**

We propose to use a Gamma distribution to model the frequencies of erroneous *k*-mers and a mixture of Gaussian distributions to model correct *k*-mers, and combine them to determine *f*_0_. To identify the two special subsets of *k*-mers, we use the *z*-score of *k*-mers which measures the number of standard deviations a *k*-mer’s frequency is from the mean. Then these statistically-solid *k*-mers are used to construct a Bloom filter for error correction. Our method is markedly superior to the state-of-art methods, tested on both real and synthetic NGS data sets.

**Conclusion:**

The *z*-score is adequate to distinguish solid *k*-mers from weak *k*-mers, particularly useful for pinpointing out solid *k*-mers having very low frequency. Applying *z*-score on *k*-mer can markedly improve the error correction accuracy.

## Background

The massively parallel next-generation sequencing (NGS) technology is revolutionizing a wide range of medical and biological research areas as well as their application domains, such as medical diagnosis, biotechnologies, virology, etc [1]. It has been shown that the NGS data is so informative and powerful that some ever thorny problems can be effectively tackled through this technology, e.g., the genome wide association study [2].

The information contained in NGS data is deep and broad, but the raw data is still error prone. Various kinds of errors exist in the raw sequencing data, including substitution, insertion and deletion. The substitution error rate can be as high as 1 to 2.5% for the data produced by the Illumina platform [3]; and the collective insertion and deletion error rate can be as high as 10 to 40% for the PacBio and Oxford Nanopore platforms [4, 5]. It has been widely recognized that correcting these sequencing errors is the first and critical step for many downstream data analyses, such as de novo genome assembly [6], variants calling from genome re-sequencing [7], identification of single nucleotide polymorphism as well as sequence mapping [3, 8]. For instance, the number of nodes of the De Bruijn graph generated from the HapMap sample NA12878 (https://www.ncbi.nlm.nih.gov/sra/ERR091571/) is 6.92 billion; however, this number can be reduced to only 1.98 billion after error correction. This reduction significantly alleviates the burden of graph manipulation.

Owing to the importance of error correction, dozens of approaches have been proposed to cope with various types of errors. Depending on the key ideas that have been used, existing approaches can be categorized into three major approaches: (i) the *k*-spectrum-based approach, including Quake [3], Reptile [9], DecGPU [10], SGA [11], RACER [12], Musket [13], Lighter [14], Blue [15], BFC [16], BLESS2 [17], MECAT [18] (ii) the suffix tree/array-based approach, including SHREC [19], HSHREC [20], HiTEC [21], Fiona [22] and; (iii) the multiple sequence alignment-based approach, including ECHO [23], Coral [8], CloudRS [24], MEC [25]. Among these approaches, the most advanced ones are the *k*-spectrum-based. It provides a very good scalability and competitive performance. Scalability is crucial for NGS data analysis since the input volume is usually huge.

**Fig. 1:**
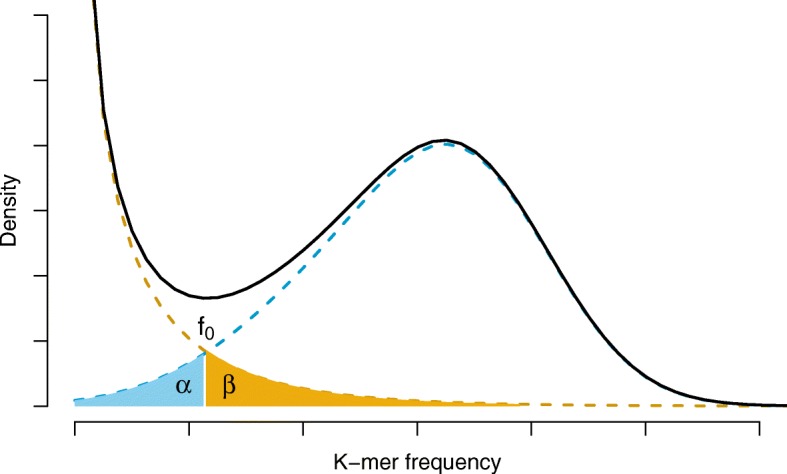
Frequency distribution of both error-free and error-containing *k*-mers for a NGS data set. The frequency distribution of erroneous *k*-mers is represented by the dash orange line, while the distribution of the correct ones is shown as the dash sky-blue line. The solid black line is the distribution of all the k-mers. The *α*-labeled area is the proportion of correct *k*-mers having frequency less than *f*_0_, while the *β*-labeled area is the proportion of erroneous *k*-mers having frequency greater than *f*_0_

The performance of *k*-spectrum-based approach heavily depends on the selection of solid *k*-mers. A solid *k*-mer is a *k*-mer frequently occurring in NGS reads. The other *k*-mers are called weak *k*-mers. A solid *k*-mer often does not contain any sequencing error, but a weak *k*-mer often contains sequencing errors. An intensively investigated problem is to find a good frequency cutoff *f*_0_ to balance the numbers of solid and weak *k*-mers, *cf.* Fig. 1. It is clear that even a very carefully determined *f*_0_ cannot tidily differentiate erroneous *k*-mers from those *k*-mers that do not contain any error bases. The reason is that there are very often a small portion of solid *k*-mers that contain errors and there are very often a tiny portion of weak *k*-mers that do not have errors, *cf.* the shaded part in Fig. 1. This discrepancy is caused by the skewed distribution of the coverage of the sequencing reads. For instance, Ross et al. [26] has reported that the coverage of GC rich and poor regions is markedly lower than the average coverage. That is, the *k*-mers from these regions very likely have low frequency, even lower than *f*_0_.

In this research, we focus on a more challenging but less-studied problem: (i) remove a small subset of solid *k*-mers that are likely to contain errors, and (ii) add a small subset of weak *k*-mers that are likely to contain no errors, into the set of solid *k*-mers. This is achieved by using *f*_0_ as well as z-score of *k*-mer, *z*(*κ*). With the purified set of solid *k*-mers, the correction performance can be much improved.

Our approach starts with counting *k*-mer frequencies by using KMC2 [27], then calculates the *z*-scores of *k*-mers. Later, the statistically-solid *k*-mers are mined by considering both frequency and *z*-score. After that, the Bloom filter is constructed by the statistically-solid *k*-mers, and the weak *k*-mers are corrected. The newly proposed approach is named as ZEC, short for *z*-score-based *e*rror *c*orrector.

## Algorithm: mining statistically-solid *k*-mers

A solid *k*-mer is conventionally defined as a *k*-mer which occurs in a data set of NGS reads with high frequency. A solid *k*-mer is usually considered error-free, and taken as the template for error correction. If a *k*-mer is not solid, then it is defined as a weak *k*-mer considered as error-containing. Existing *k*-mer-based approaches use a frequency cutoff, *f*_0_, to identify solid and weak *k*-mers from NGS reads, e.g., BLESS2 [17], Musket [13], and BFC [16]. The main difference of these methods is how the *f*_0_ is determined.

In fact, a solid *k*-mer is not definitely error-free. Sometimes, it may contain errors with a small chance. It is also true for the weak *k*-mers — a weak *k*-mer can be absolutely error-free. The reason that a solid *k*-mer is not always correct is that the coverage is not under uniform distribution. Thus the cutoff *f*_0_ itself is unable to perfectly distinct correct *k*-mers from erroneous *k*-mers; *cf.* the part labeled as *α* and *β* in Fig. 1. However, the purpose of the research is to obtain correct *k*-mers as many as possible.

In this study, we present a time and memory efficient algorithm to purify the solid *k*-mer set as well as the weak *k*-mer set, so that more correct *k*-mers can be identified.

Let *R* be the input set of NGS reads, and *K* be the set of *k*-mers contained in *R*. To determine whether a *k*-mer, say *κ*, of *K* is correct or not, the following metrics are examined:

- *f*(*κ*), the frequency of *κ*;
- *z*(*κ*), the z-score of *κ*.

### Calculating *f*(*κ*)

The straightforward approach to determine *f*(*κ*) is as follows: (i) scan each read *r* of *R* from the beginning to the end; (ii) sum over the occurrence that *κ* appears. Then the summation is *f*(*κ*). This approach works for one *k*-mer, but it cannot be applied to all the *k*-mers simultaneously as the number of *k*-mers can be very large, demanding a huge size of memory.

In this study, we make use of the k-mer counting algorithm, KMC2 [27], to solve this problem. KMC2 can remarkably reduce the memory usage because: (i) it is disk-based; (ii) it uses (*k*,*x*)-mer; and (iii) it applies the minimizer idea to deal with *k*-mer.

### Computing *z*(*κ*)

Given a *k*-mer *κ*, we define the neighbor of *κ*, *N*(*κ*), as

$${N}(\kappa) = \left\{\kappa^{\prime}: {D}\left(\kappa, \kappa^{\prime}\right) \leq d_{0}, \kappa^{\prime} \in {K} \right\}, $$

where *D*(*κ*,*κ*^′^) is the edit distance between *κ* and *κ*^′^, and the *d*_0_ is the predefined maximum distance. The default value of *d*_0_ is 1 as used in this study, but user can adjust this value to any reasonable integer.

The *k*-mer cluster centered at *κ* is defined as

$${C}(\kappa) = \{\kappa\}\cup {N}(\kappa), $$

and the set of frequencies associated with these *k*-mers is defined as

$${F}(\kappa) = \{f(\kappa): \kappa \in {C}(\kappa)\}. $$

The *z*-score of *κ*, *z*(*κ*), is computed by

$$z(\kappa) = \frac{f(\kappa) - \mu}{\sigma}, $$

where *μ* is the averaged frequency of *F*(*κ*) and *σ* is the standard deviation of *F*(*κ*).

It is straightforward to calculate the *z*-score of each *k*-mer given the frequency of the *k*-mer as well as that of its neighbor that have been determined by the aforementioned approach.

### Determining *f*_0_

Unlike existing approaches that determining solid *k*-mers based on their frequency only, we examine their *z*-scores as well.

Traditionally, an optimal *f*_0_ is used to distinct weak and solid *k*-mers, which is determined as the count minimizing misclassification rates (see misclassified parts labeled as *α* and *β* in Fig. 1). To learn the optimal value, we model the frequency of erroneous *k*-mers by a Gamma distribution *P*_*G*_(*X*), and those correct ones by a mixture of Gaussian distributions *P*_*N*_(*X*). A Gamma distribution is defined as:

$$P_{G}(X=x; k, \theta) = \frac{1}{\Gamma(k)\theta^{k}}x^{k-1}e^{-\frac{x}{\theta}} $$

where *k* accounts for the shape of the distribution, *θ* is for the scale of the distribution, i.e., how the data spread out, *Γ*(*k*) is the Gamma function evaluated at *k*; *cf*. the dash sky-blue line in Fig. 1. While a mixture of Gaussian distributions is

$$P_{N}(X=x; \pi, \mu, \sigma) = \sum\limits_{i=1}^{K} \pi_{i} \cdot \mathcal{N}(\mu_{i}, \sigma_{i}), $$

where *π*_*i*_ is the mixture parameter, *μ*_*i*_ and *σ*_*i*_ represent the mean and standard deviation of the component *i*, and *K* is the number of Gaussian components. In this study, *K* is set as 2, with one accounting for *k*-mers that are from GC rich or poor regions, and the other for the rest correct *k*-mers.

The two distributions are estimated by using EM algorithm based on the frequencies of *k*-mers. An example of the two distributions are shown in Fig. 1, i.e., the sky-blue dash line and the orange dash line. Based on the two distributions, we can determine the threshold *f*_0_, such that it can minimize the area marked as *α* and *β*. Note that, the threshold *f*_0_ determined in this way may not be the intersection point of the two density functions.

### Mining solid *k*-mers

It is clear that the optimal *f*_0_ cannot perfectly distinct the solid *k*-mers from the weak *k*-mers. Taking Fig. 1, the *k*-mers marked by *α* will be wrongly corrected although they do not have errors but just because their frequencies are lower than *f*_0_; likely, the ones marked by *β* will keep unchanged although they have errors because they have high frequency. To further refine the *purity* as well as the *completeness* of solid k-mers, we borrow the statistical idea of using *z*-score to solve the problem. The *purity* is defined as

$$\text{purity}=1-p^{2}_{\text{correct}}-p^{2}_{\text{erroneous}}~, $$

where *p*_correct_ is the proportion of correct *k*-mers in the solid *k*-mers, and *p*_erroneous_ is the proportion of erroneous *k*-mers in the solid *k*-mers. The *completeness* is calculated as

$$\text{completeness}=N^{\text{solid}}_{\text{correct}}/N_{\text{correct}}~, $$

where $N^{\text {solid}}_{\text {correct}}$ is the number of correct *k*-mers in the solid *k*-mers, and *N*_correct_ is the total number of correct *k*-mers.

The *z*-score as well as the frequency are collectively incorporated into solid *k*-mer identification through the following two situations:

- If *f*(*κ*)<*f*_0_ and *z*(*κ*)≥*z*_0_, then *κ* is removed from the weak *k*-mers and added to the solid *k*-mers, i.e., increases the completeness.
- If *f*(*κ*)≥*f*_0_ and $z(\kappa) < z^{'}_{0}$, then *κ* is removed from the solid *k*-mers and added to the weak *k*-mers, i.e., improves the purity.

The *f*_0_ is the minimum frequency that has been determined, while the *z*_0_ and $z^{'}_{0}$ are the maximum *z*-score and minimum *z*-score for weak *k*-mers and solid *k*-mers, respectively.

The *z*_0_ and $z^{'}_{0}$ are learned from the *z*-score distribution automatically. To obtain the optimal *z*_0_, the z-scores of the *k*-mers having frequency less than *f*_0_ are collected. Later, the distribution of these *z*-scores is estimated and *z*_0_ is set as the value having the lowest density between two peaks (viz. the trough of the bimodal; see results for more details). Analogously, $z^{'}_{0}$ is determined on the *z*-scores of *k*-mers having frequency greater than *f*_0_.

## Methods

Our error correction model contains two main steps: (i) build Bloom filter from solid *k*-mers and; (ii) correct errors in weak *k*-mers by the Bloom filter.

### Build bloom filter

Bloom filter [28] is a probabilistic data structure that can check whether an item is contained in a set of items with very frugal memory consumption. Instead of storing each item as is, the Bloom filter maps the item into several bits of a bit vector. Each bit can be reused by many items, and the mapping is achieved by hash functions. To check whether an item exists in a set of items, one only need to check whether all the mapped bits are “1”s. In case any one of them is “0”, it indicates that the item is definitely not contained in the set. Since each bit can be reused, it is possible that an item is not contained in the set but all of its mapped bits are “1”s. The probability that it happens is false positive rate. The relation between the number of hash function *h*, the false positive rate *p*, the size of the bit vector *n*, and the actual number of elements *m* is

$$p = \left(1-\left(1-\frac{1}{n}\right)^{hm}\right)^{h} \approx \left(1-e^{-h\frac{m}{n}}\right)^{h}. $$

In our study, *m* is the number of solid *k*-mers that have been determined from all the *k*-mers by means of the aforementioned algorithm. Per existing approaches, *p* is set to 1%. One can also tune *p*, *h* and *n* to fit the real hardware limitations.

It has been reported that the Bloom filter has been successfully used to correct NGS errors, such as BLESS2 [17] and BFC [16]. The major difference between our model and the existing models is that we dedicate to efficiently refine the solid *k*-mers that are used to construct Bloom filter, which directly improves the error correction performance in theory. Note that, the solid *k*-mers play the key role in error correction, as all the rest *k*-mers (viz. the weak *k*-mers) are to be corrected based on the *solid* ones.

**Fig. 2:**
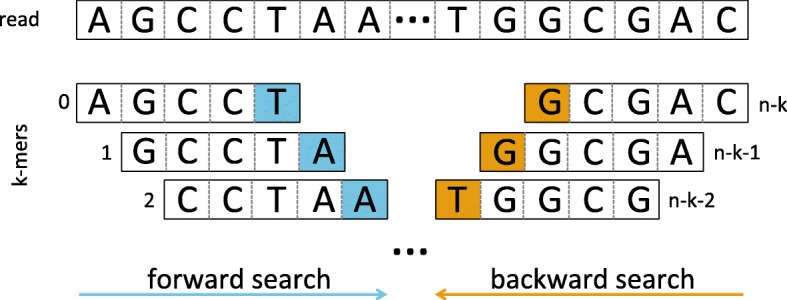
Illustration of the forward and backward search to correct sequencing errors. The forward search starts from the first *k*-mer to the last *k*-mer. At each step the last base of the *k*-mer is substituted by its alternatives to check the solidity. Inversely, the backward search starts from the last *k*-mer to the first *k*-mer. On the contrary to the forward search, the first base of the *k*-mers are altered other than the last one

Figure 2 illustrates the forward search and backward search.

### Correct errors

By using Bloom filter, the errors contained in each read can be correct as follows: (i) check the existence of each *k*-mer of the read from the beginning to the end sequentially. (ii) partition the *k*-mers into groups that each group contains only solid *k*-mers or weak *k*-mers, deemed as solid group *G*_*s*_ or weak group *G*_*w*_, respectively. The order of the groups is kept according to their appearance in the read. (iii) correct the errors causing the weak group *G*_*w*_ according to the following situations:

1. If *G*_*w*_ is the first group and there exists a successive group *G*_*s*_ that is solid, we iteratively change the first base of each *k*-mer of *G*_*w*_ to its alternatives and check the existence of the *k*-mers against the Bloom filter. Once there exist a solution that makes all the weak *k*-mers solid, the amendment of the bases is accepted, thus the correction of the error. This process is applied to the *k*-mers of *G*_*w*_ from the last one to the first one. In case the number of *k*-mers contained in *G*_*w*_ is less than a predefined value, say *τ*, the processive solid *k*-mers that are extended from the corrected *k*-mers will be generated until the total number of *k*-mers in *G*_*w*_ is *τ*. If this criterion cannot be satisfied, the solution is abandoned. On the other hand, if *G*_*s*_ does not exist, we will alter the bases to their alternatives of all the *k*-mers iteratively until a solution that make all the *k*-mers solid can be found.
2. If *G*_*w*_ has a solid processive group *G*_*s*_ and a solid successive group $G_{s}^{'}$, we substitute the last base of each *k*-mer in *G*_*w*_ by its alternatives from the first *k*-mer to the last *k*-mer, namely the forward search. Solutions that make all the *k*-mers solid till the current substitution are recorded. Similarly, the backward search is conducted on the first base of the *k*-mers from the last one to the first one. A solution is accepted if the forward search and the backward search meet and the *k*-mers contained in both of them are solid. In case the number of *k*-mers in *G*_*w*_ is less than *k*, we will only alter the last base of the first *k*-mer.
3. If *G*_*w*_ is the last group and there exists a solid processive group *G*_*s*_, we will apply the backward search to obtain the solution. Analogously to the first situation, if the number of *k*-mers of *G*_*w*_ is less than *τ*, we will extend the *k*-mers toward their downstream until the number is satisfied. In case *G*_*s*_ does not exist, it is the same as the second part of the first situation, thus the same approach is applied.

## Results

### Datasets

We collected six data sets to test the performance of our proposed method in comparison with the state-of-art methods. Four of the six data sets are the NGS reads produced by the Illumina platform, including Staphylococcus aureus (S. aureus), Rhodobacter sphaeroides (R. sphaeroides), Human Chromosome 14 (H. chromosome 14) and Bombus impatiens (B. impatiens). These data sets are the gold standards used by GAGE [6] for NGS data analysis. Besides these real data sets, two synthesized data sets have been generated by using ART [29] based on the genomes H. chromosome 14 and B. impatiens. The two synthetic data sets contain exactly the same number of reads as the real ones. They are included because the ground truth of the synthesized errors are known, i.e., the positions of the errors as well as their bases are available. On the contrary, such information is unavailable for the real data sets. Typically, the raw reads of the real data sets are mapped to the corresponding reference, and those mapped are kept for performance evaluation. Although this is arguable as various deleterious situations can emerge from the mapping, e.g., unmapped reads, multi-mapped reads, wrongly mapped reads, it is necessary to carry out the mapping as only in this way can we perform the evaluation directly. This is another reason that the synthetic data should be included. Details of these data sets are shown in Table 1.

**Table 1:** The data sets that are used for evaluating the performance of error correction models

Data set	Genome name	Genome size (bp)	Error rate (%)	Read length (bp)	Coverage	Number of reads	Insert length	Is sythetic
R1	S. aueus	2,821,361	1.28	101	46.3 ×	1,294,104	180	No
R2	R. sphaeroides	4,603,110	1.08	101	45.0 ×	2,050,868	180	No
R3	H. chromosome 14	88,218,286	0.52	101	41.8 ×	36,504,800	155	No
R4	B. impatiens	249,185,056	0.86	124	150.8 ×	303,118,594	400	No
S1	H. chromosome 14	88,218,286	0.97	101	41.8 ×	36,504,800	180	Yes
S2	B. impatiens	249,185,056	0.98	124	150.8 ×	303,118,594	400	Yes

### Performance evaluation

The error correction performance is evaluated through the widely accepted procedure implemented by [30]. Metrics that are considered include *gain*, *recall*, *precision* and per base error rate (pber). Gain is defined as (*TP*−*FP*)/(*TP*+*FN*), recall is *TP*/(*TP*+*FN*), precision is *TP*/(*TP*+*FP*) and pber is *N*^*e*^/*N*, where *TP* stands for the number of corrected bases that are truly erroneous bases, *FP* represents the number of corrected bases that are not sequencing errors intrinsically, *FN* is the number of erroneous bases that remain untouched, *N*^*e*^ is the number of erroneous bases and *N* is the total number of bases. Among these metrics, *gain* is the most informative.

All experiments are carried out on a cluster having eight Intel Xeon E7 CPUs and 1Tb RAM. Each CPU has eight cores.

*Overall Performance of ZEC*. The experimental results of ZEC are presented in Table 2. ZEC performs well on both of the real data sets and the synthetic data sets. Comparing the performance on H. chromosome 14 and B. impatiens, ZEC has a much better performance on S. aueus and R. sphaeroides. This is consistent with our understanding that the genomes of the former two data sets are much more complicated than the latter two, where the errors introduced in complicated genomes are more difficult to correct.

**Table 2:** Error-correction performance comparison between ZEC, Lighter, Racer, BLESS2, Musket, BFC, SGA and MEC

Data	Corrector	Gain	Reca	Prec	Pber(%)
R1	ZEC	0.908	0.912	0.996	0.102
	Lighter	0.839	0.845	0.994	0.163
	Racer	0.760	0.822	0.929	0.190
	BLESS2	0.189	0.409	0.650	0.879
	Musket	0.499	0.628	0.830	0.448
	SGA	0.746	0.815	0.922	0.202
	BFC	0.753	0.817	0.927	0.196
	MEC	**0.909**	0.911	0.998	0.102
R2	ZEC	**0.584**	0.663	0.894	0.537
	Lighter	0.226	0.329	0.762	1.076
	Racer	0.364	0.450	0.839	0.780
	BLESS2	0.318	0.405	0.806	0.890
	Musket	0.265	0.364	0.786	0.984
	SGA	0.331	0.423	0.822	0.843
	BFC	0.306	0.400	0.811	0.893
	MEC	0.570	0.631	0.912	0.541
R3	ZEC	**0.802**	0.923	0.884	0.087
	Lighter	0.445	0.764	0.706	0.256
	Racer	0.562	0.814	0.764	0.196
	BLESS2	0.130	0.641	0.556	0.438
	Musket	0.533	0.802	0.749	0.211
	SGA	0.567	0.818	0.765	0.194
	BFC	0.603	0.833	0.783	0.176
	MEC	0.788	0.852	0.930	0.117
R4	ZEC	**0.746**	0.833	0.905	0.137
	Lighter	0.126	0.408	0.591	0.688
	Racer	0.313	0.541	0.703	0.484
	BLESS2	-0.517	0.018	0.003	0.862
	Musket	0.502	0.660	0.807	0.320
	SGA	0.542	0.690	0.823	0.289
	BFC	0.195	0.457	0.636	0.607
	MEC	0.705	0.806	0.889	0.201
S1	ZEC	**0.918**	0.935	0.982	0.056
	Lighter	0.791	0.851	0.934	0.130
	Racer	0.882	0.916	0.964	0.071
	BLESS2	0.634	0.740	0.875	0.243
	Musket	0.819	0.871	0.944	0.111
	SGA	0.810	0.865	0.940	0.117
	BFC	0.866	0.903	0.961	0.081
	MEC	0.899	0.916	0.982	0.063
S2	ZEC	**0.853**	0.894	0.956	0.109
	Lighter	0.058	0.329	0.548	0.891
	Racer	0.168	0.408	0.630	0.720
	BLESS2	0.311	0.509	0.719	0.543
	Musket	0.232	0.453	0.672	0.636
	SGA	0.075	0.342	0.562	0.862
	BFC	0.751	0.822	0.920	0.157
	MEC	0.849	0.887	0.959	0.122

The numbers in bold face are the best gain achieved for each data set

**Fig. 3:**
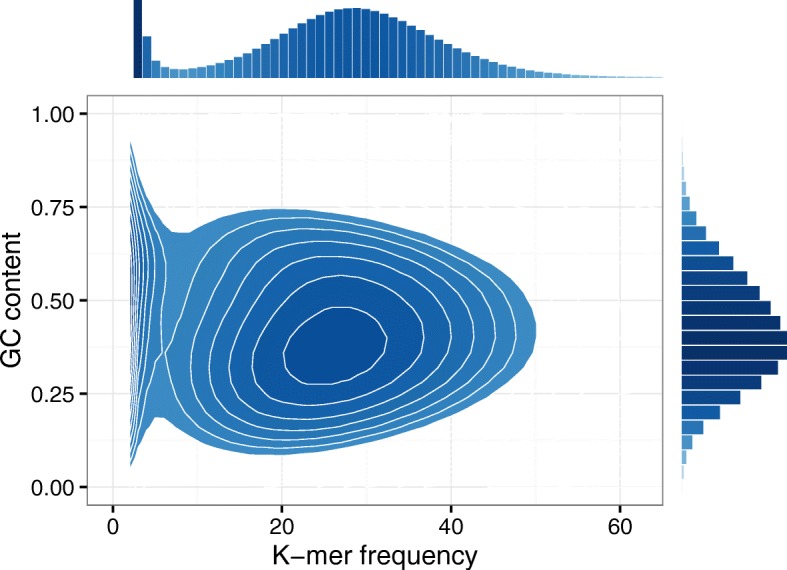
A relation between *k*-mer frequency and GC-content. The bottom left panel shows the smoothed scatter plot between *k*-mer frequency and GC-content, the top left is the distribution of *k*-mer frequency, and the bottom right is the distribution of GC-content. It is clear that GC-content *k*-mers have relatively low frequency. The data shown in this example is obtained from the H. chromosome 14 with *k*-mer size of 25

*Relation with GC-content*. A previous study by Ross et al. [26] shows that the GC-content (GC poor and GC rich) regions have direct influence on the low sequencing coverage of NGS data. Hence, the *k*-mers obtained from the reads sequenced from these regions are more likely to be treated as weak. Figure 3 highlights an example of the relation between GC-content (GC poor and GC rich) and *k*-mer frequency derived from H. chromosome 14. It can be seen that the *k*-mers having a low frequency can spread out wider than those having a high frequency, and the wide range is coincident with the GC content. This result is in accordance with the performance shown in Table 2, meanwhile it also consolidates our intuition that refining the set of solid *k*-mers is necessary, particularly for the subset of *k*-mers that have a low frequency. More importantly, it empirically supports our idea of using mixture model to treat solid *k*-mers and weak *k*-mers separately.

*Comparison with State-of-the-art*. The performance of ZEC is much superior to the state-of-the-art methods, including Lighter [14], Racer [12], BLESS2 [17], Musket [13], SGA [11], BFC [16]. See Table 2. ZEC markedly outperforms the existing error correctors in terms of the most informative evaluation metric—*gain*. For instance, on the dataset R4, the gain of ZEC is 0.746, while the best performance produced by the other methods is 0.705. For the synthetic datasets, ZEC also has higher gain than other methods. For example, on the dataset S2, the gain of ZEC is 0.853, while the best and worst gain generated by the other methods are 0.849 and 0.058, respectively. The lowest average per-base error rate of ZEC also consolidates its effectiveness.

### Distinguishbility of z-score

**Fig. 4:**
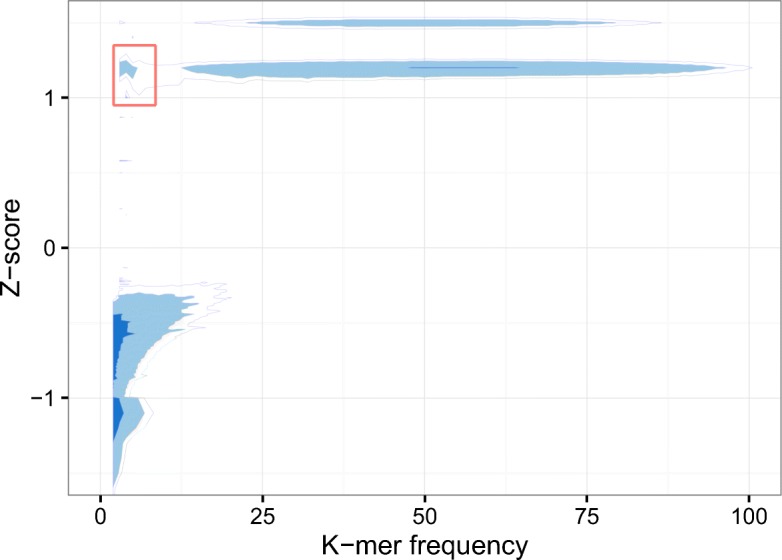
A relation between *z*-score and *k*-mer frequency. The level of shade represents the density of the distribution. The darker the color is, the more *k*-mers are presented. The frequencies of the *k*-mers highlighted in the red box are less than nine, which are very likely to be treated as weak for all existing *k*-mer based approaches. However, the very high *z*-score reflects that they should be treated as solid *k*-mers. The data shown here is obtained from B. impatiens with *k*-mer size of 25

The key to the performance improvement is the idea of using *z*-score for identifying the two special subsets of *k*-mers from the sets of solid *k*-mers and weak *k*-mers. An example of *z*-score distribution pertaining to *k*-mer frequency is shown in Fig. 4, which is derived from B. impatiens. The highlighted *k*-mers shown in the figure have relatively low frequencies—less than 9, while the *z*-scores are pretty high—greater than 1. Interestingly, almost all the solid *k*-mers (the top right region) have the similar level of *z*-scores comparing to these highlighted ones. These observations indicate that the highlighted k-mers are very likely to be correct *k*-mers instead of erroneous *k*-mers although their frequencies are very low. The *z*-score distribution pertaining to the other three real data sets has similar patterns compared to the one shown here.

**Fig. 5:**
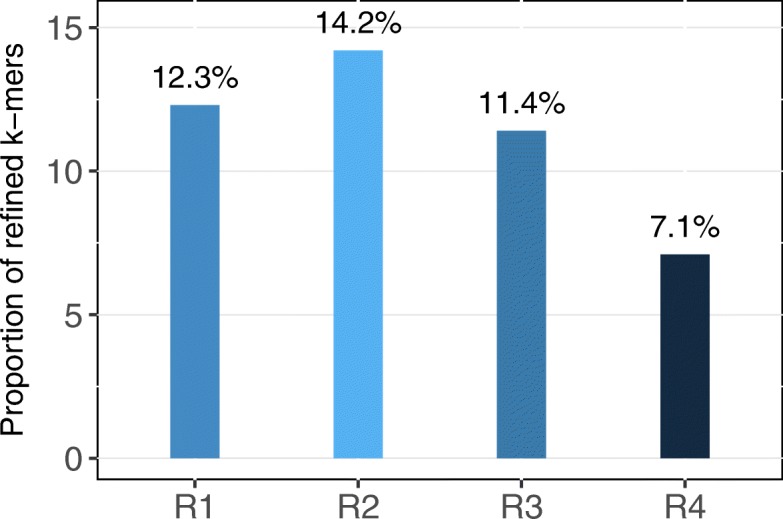
The proportion of *k*-mers refined by *z*-score. The refinements come from two folds: weak *k*-mers having high *z*-score (moved to the solid *k*-mer set), and solid *k*-mers having low *z*-score (excluded from the solid *k*-mer set)

By exploring the four real data sets, we found that the proportion of *k*-mers that can be refined comparing to the solely frequency determined *k*-mers are 12.3%, 14.2%, 11.4%, 7.1% for the real data R1, R2, R3 and R4, respectively; see Fig. 5. These refinement are the major contributions of the performance improvement.

### Efficiency of *z*-score calculation

Calculating z-score of *k*-mers is not trivial for very large data sets, as the *k*-mers and their frequencies are usually too large to be hold by a main memory of a moderate computer. We designed a novel algorithm and solved this problem. The efficiency of the algorithm in terms of the memory usage and running speed are studied.

**Fig. 6:**
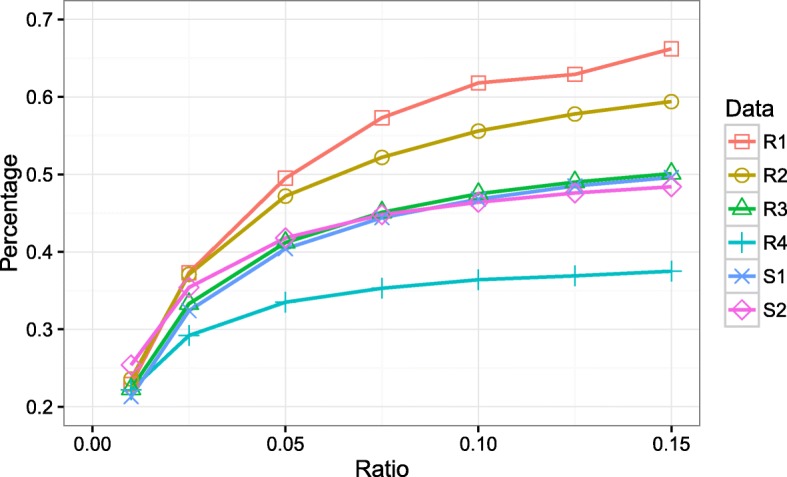
Memory saving analysis on the six data sets. The x-axis shows the memory saving ratio between the size of real memory allocation and raw input, while the y-axis shows how much proportion of an input held by a bit vector

Figure 6 shows the relation between memory saving ratio and the percentage of input (*k*-mers as well as their frequencies) that can be held by only one bit vector. The memory saving ratio is calibrated as the ratio between the real memory allocation and the input data volume. For instance, the ratio of 0.01 pertaining to the data R4 means that the allocated memory is one percent of the input size of R4. That is, 70Mb memory is allocated for holding the 6.97Gb data. It is promising that, with one percent memory allocation, around 22 percent input data can be hold by only one bit vector. When the memory allocation increased to 2.5 percent, 30 percent input data and even more can be held by one bit vector. Obviously, keep increasing the size of allocated memory does not guarantee the linear scale of holding the input data. Based on the experiments, we set the memory allocation ratio to 2.5 percent through the whole study. Typically, three bit vectors are constructed for holding all the input. Note that, the size of bit vector decreases along with the reduced size of input. The ratios between the input and the total allocated memory are 20.0, 13.4, 7.0 and 7.9 for the four real datasets, respectively.

Regarding the running speed, this algorithm is linearly scaled. Since locating each *k*-mer in a bit vector is O(1) pertaining to time complexity by using hash, this algorithm is pretty fast. For instance, based on our computing power, it only takes 387 s to construct the bit vectors and calculate the z-scores of all the *k*-mers of R4—the largest data set.

Since a Bloom Filter has false positives, this may cause the *z*-score of a *k*-mer different from its genuine value. However, the false positive rate is pretty small, usually less than 1%, thus this impact can be neglected.

## Discussion

Our model effectively pinpoints out correct *k*-mers having low frequency, achieving an improvement of 11.25% on weak *k*-mers. However, some issues still remain further exploration, including neighbor inclusion and neighbor retrieval.

Neighbor inclusion means how neighbor *k*-mers are determined given a *k*-mer of interest, say *κ*. Our current approach takes *k*-mers having edit distance of 1 as neighbors of *κ*, but there still has a small chance that a true neighbor having edit distance larger than 1. Suppose the error rate is *e*, the probability of a *k*-mer having exactly one error is *k*·*e*(1−*e*)^*k*−1^/*k*·*e*=(1−*e*)^*k*−1^. When *e*=1% and *k*=1, the probability is (1−0.01)^31−1^=73.97*%*. That been said, about 26% real neighbors are excluded. However, even extending the minimum edit distance from 1 to 2 significantly elongates running time. This is because the number of candidate *k*-mers increases from 3∗*k* to 3∗*k*∗3∗(*k*−1).

Neighbor retrieval is another issue to be considered. Usually, the size of counted *k*-mers is too large to fit into a main memory. Hence, a more sophisticated approach is required to solve this problem. We use Bloom Filter to overcome the limitation. For *k*-mers having small count, say 5, we use classical Bloom Filters to save them, each Bloom Filter saves *k*-mers having the same count. For *k*-mers having large count, we use coupled-Bloom Filter to save them. One Bloom Filter for *k*-mer encoding, while the other is for count representation. This approach significantly reduces memory usage while achieving constant time complexity of *k*-mer retrieval. However, it may cause false positives although the probability is small. Hence, more effort is required to handle this problem.

## Conclusions

We have proposed a novel method for correcting the NGS errors. The novel idea is the use of statistically-solid *k*-mers to construct the Bloom filter. These *k*-mers are mined from all the *k*-mers of a NGS data set by considering both their frequency and *z*-score, particular the latter one that can effectively fishing out the solid *k*-mers having low frequency. Pinpointing out such *k*-mers has been a very challenging problem. The experimental results show that our approach markedly outperforms the existing state-of-the-art methods in terms of error correction performance.
